# Running Plus Strength Training Positively Affects Muscle Strength and Quality in Both Younger (Below 50 Years Old) and Older (Above 50 Years Old) Women

**DOI:** 10.3390/geriatrics9050127

**Published:** 2024-10-04

**Authors:** Lavínia Vivan, Vinícius Ribeiro dos Anjos Souza, Aldo Seffrin, Claudio Andre Barbosa de Lira, Rodrigo Luiz Vancini, Katja Weiss, Beat Knechtle, Marilia Santos Andrade

**Affiliations:** 1 Postgraduate Program in Translation Medicine, Federal University of São Paulo, São Paulo 04023-062, Brazil; laviniavivan@gmail.com (L.V.); viniciusribeiro89@hotmail.com (V.R.d.A.S.); netoseffrin@gmail.com (A.S.); 2 Human and Exercise Physiology Division, Faculty of Physical Education and Dance, Federal University of Goiás, Goiás 74690-900, Brazil; claudioandre@ufg.br; 3 Center for Physical Education and Sports, Federal University of Espírito Santo, Espírito Santo 29075-810, Brazil; rodrigoluizvancini@gmail.com; 4 Institute of Primary Care, University of Zurich, CH-8006 Zurich, Switzerland; katja@weiss.co.com; 5 Department of Physiology, Federal University of São Paulo, São Paulo 04023-062, Brazil; marilia1707@gmail.com

**Keywords:** aging, isokinetic, physical activity, sarcopenia

## Abstract

Background/Objectives: Sarcopenia is a muscular disease characterized by loss of muscular strength and function, affecting mainly women, and associated with increased mortality risk. The aim of this study was to compare active women with inactive women of different age groups regarding muscle mass, strength, and muscle quality. Methods: This study included 147 women (85 runners and 62 inactive), divided into <50 and ≥50 years old. Participants were evaluated for knee flexor and extensor peak torque (PT), body composition, and training habits. Results: For knee extensor muscles, there was an age group effect (F(2.146) = 40.5; *p* < 0.001) on absolute PT (Nm); an age group effect (F(2.146) = 44.1; *p* < 0.001) and a physical activity group effect (F(2.146) = 113.0; *p* < 0.001) on PT adjusted by body mass (Nm/kg); and an age group effect (F(2.146) = 36.9; *p* < 0.001) and a physical activity group effect (F(2.146) = 6.1; *p* = 0.014) on PT adjusted by lean mass (Nm/kgLM). There was no interaction effect. Conclusion: In both age groups, active women had greater strength and higher muscle quality than inactive women, but the difference in strength, muscle mass, and muscle quality between younger and older women were the same among runners and inactive women.

## 1. Introduction

Sarcopenia is a skeletal muscle disorder (muscle insufficiency) characterized by a gradual loss of strength or low muscle quality [1,2]. Although it is typically associated with older people, muscle mass decrease starts around 40 yrs old [3], which is quite concerning given that muscular weakness has been associated with several adverse outcomes, including falls, functional decline, frailty, and mortality [4]. Given the importance of this problem, sarcopenia has been extensively studied to improve the knowledge of its diagnosis, treatment, and prevention [1]. Considering the risk factors for sarcopenia, the most commonly cited modifiable risk factors include poor diet and low physical activity [5].

The effects of low physical activity levels are especially concerning for women because they are less active than males [6] and have a longer life expectancy at birth [7]. Therefore, they may live longer, exposing them to the adverse effects of sarcopenia. A previous study showed a higher incidence of sarcopenia in women than in men (17% vs. 12%, respectively) [3].

Despite the compelling evidence that physical activity increases muscle mass and strength [8,9], the aging process negatively affects the skeletal muscles even in active people. It has been demonstrated in sedentary men that a muscle mass loss of approximately 1.4% each year occurs after the age of 50 yrs old [10], and, in some cases, a loss of about 50% occurs by the eighth to ninth decade of life [11]. However, it is unknown if active individuals have the same rate of muscle strength loss as sedentary individuals.

The loss of muscle mass is undeniably important, but the loss of strength and function appears to be higher than the loss of mass, at least in sedentary individuals [12]. This difference may be partly due to muscle quality impairment, such as a loss of muscle strength per unit muscle mass [13], which has previously been observed for sedentary individuals, but not athletes. Muscle mass, strength, and quality may be precisely assessed using modern imaging methods, such as dual-energy X-ray absorptiometry (DXA) or dynamometry devices, such as isokinetic dynamometry [13].

Regarding the physical activity type, running is a widely practiced activity, which is time-efficient, easily accessible, and relatively low in cost [14]. The improvement in cardiorespiratory conditioning is among the main health benefits of running [15]; however, its effects on muscular strength, mass, and quality are poorly studied. Therefore, the present study aimed to compare active women performing running with inactive women in different age groups (under and over 50 yrs old) for muscle mass, strength, and quality. We hypothesized that in both age groups, muscle strength, mass, and quality would be greater in the runners’ group than in the inactive group and the strength difference between women under and over 50 yrs old would be similar in both active and inactive groups.

## 2. Materials and Methods

### 2.1. Ethical Approval

All the experimental procedures presented in the study were approved by the Human Research Ethics Committee of the Federal University of São Paulo (approval number 4.354.386). Participants were informed about the study’s aims, risks, and benefits and signed a consent form. All participants were guaranteed their privacy and confidentiality.

### 2.2. Participants

One hundred and forty-seven women participated in the study. There were 85 active participants (age: 45.8 ± 14.1 yrs old, height: 161.1 ± 6.3 cm, body mass: 59.9 ± 7.9 kg, and body mass index (BMI): 23.1 ± 3.1 kg/m^2^) and 62 inactive participants (age: 48.8 ± 9.8 yrs, height: 160.2 ± 5.7 cm, body mass: 82.2 ± 11.8 kg, and BMI: 31.9 ± 4.3 kg/m^2^). They were recruited through running coaches and social media outreach. Both active and inactive groups were divided into two age groups: <50 yrs and ≥50 yrs. The active group under 50 yrs was (*n* = 51), the active group 50 yrs or above was (*n* = 34), the inactive group under 50 yrs was (*n* = 33), and the inactive group 50 yrs or above was (*n* = 29). The decision to divide the age groups by 50 yrs was made because the decline in strength becomes more evident after this age [10,16].

The inclusion criteria for the active groups were women who had participated in running for more than 3 yrs. According to the International Physical Activity Questionnaire (IPAQ), all volunteer women in the inactive group should be classified as inactive [17]. This questionnaire was validated for the Portuguese language, and it has acceptable measurement properties for estimating physical activity levels [17,18]. The level of physical activity can be classified into 5 categories according to answers to the IPAQ. The categories are very active (vigorous activities 5 days/week and ≥30 min per session or vigorous activities ≥3 days/week and ≥20 min per session plus moderate activities ≥ 5 days/week and ≥30 min per session); active (vigorous activities ≥ 3 days/week and ≥20 min per session, moderate activities ≥5 days/week and ≥30 min per session, or any combined activity ≥5 days/week and ≥150 min/week such as walking plus moderate plus vigorous); irregularly active A (insufficient to be classified as active because it does not comply with the recommendations regarding frequency or duration); irregularly active B (insufficient to be classified as irregularly active A because it does not comply with either the frequency or duration recommendations); and inactive (those who do not perform any physical activity for at least 10 continuous minutes during the week) [18].

Participants ranged in age from 20 to 70 yrs old. The study excluded women with chronic diseases that impair muscle function, such as neurological, muscular, cardiovascular, and respiratory diseases. Participants who experienced pain or discomfort during the isokinetic muscle assessment would be excluded from the study. However, nobody was excluded from the study.

### 2.3. Study Design

This was a cross-sectional study. In the morning, participants went to the Exercise Physiology Laboratory for an isokinetic strength test and body composition evaluation. They were instructed to dress comfortably and without metal objects. Participants completed a questionnaire on their training habits and general health characteristics before the tests.

Body height and mass were assessed using a calibrated wall stadiometer and an electronic scale (Filizola^®^, São Paulo, Brazil), respectively. The measurements were recorded to the nearest 0.1 cm and 0.1 kg, respectively.

### 2.4. Experimental Procedures

#### 2.4.1. Questionnaires

The first questionnaire included the following questions: How long have you been performing running training? How much running training do you perform every week (hours per week)? How much strength training do you undertake every week (hours per week)? The second questionnaire was the IPAQ [17].

#### 2.4.2. Isokinetic Muscle Assessment

The isokinetic strength test is a reliable and reproducible test for evaluating knee muscle strength. Therefore, a single testing session is required to obtain reliable results [19]. The tests were performed using a Biodex System 3 isokinetic dynamometer (Biodex Medical System, Shirley, NY, USA) to evaluate the isokinetic concentric strength of the individuals’ dominant lower limb, which was determined by asking which limb they preferred to use when kicking a ball. All participants warmed up for 5 min before the isokinetic test on a cycle ergometer (25 W) for the lower extremities (Cybex Inc., Ronkonkoma, NY, USA). The participants sat with their hips flexed at approximately 85° and were fastened across the trunk and waist to the isokinetic dynamometer. The strength of the knee flexor and extensor muscles was assessed through 90° of range of motion. Gravity correction was performed with the knee fully extended. Before the test, the participants were given three trials at submaximal effort with a gradually increasing load (50–75% of maximum effort) to familiarize themselves with the range of motion and accommodating resistance of the dynamometer. Then, in concentric mode, they performed one set of five repetitions at maximum effort at angular speeds of 60°/s. The coefficient of variance was used to assess the quality of the data produced by the isokinetic dynamometer. Data with a coefficient of more than 10% variance were disregarded as valid for analysis, and another test was performed after 10 min. Throughout the test, participants were provided continuous verbal encouragement to help them achieve their fastest and strongest contractions. All participants were tested by a trained and experienced examiner using the isokinetic dynamometer. Peak torque (PT) in Nm and PT adjusted to total body mass (Nm/kg) were used as outcome measures for knee flexor and extensor muscles. To infer muscle quality, PT values were also adjusted to the lower-limb lean mass (Nm/kgLM) using the outcomes determined by the method indicated below (DXA).

#### 2.4.3. Body Composition Assessment

Whole-body bone mineral density (g/cm^2^), whole-body lean mass (kg), lower-limb lean mass (kg), and whole-body fat mass (%) were measured using a DXA (software version 12.3, Lunar DPX, Madison, WI, USA) [20]. DXA provides accuracy across a wide range of body sizes and types, which is critical for our study. [20] Moreover, it involves minimum radiation exposure, which has been considered safe [20,21]. To perform the test, participants were evaluated in a supine position, and they were centrally aligned in DXA, with 10 cm between the feet and 5 cm between the hands and trunk. All the tests were performed by the same experienced examiner.

### 2.5. Statistical Analysis

The values are presented as means, standard deviations, and effect sizes. A significant effect size of 0.25, a significance level of 0.05, and a power of 0.80 were used to calculate the sample size. According to the study results, a total sample size of 128 participants (32 in each group) was required. According to the Shapiro–Wilk test, all data had a normal distribution, and homogenous variances were confirmed using the Levene test.

Physical activity group (inactive vs. active); age group (<50 vs. ≥50 yrs); and interaction effects on absolute values for PT (Nm), total body mass-adjusted PT (Nm/kg), and lean mass-adjusted PT (Nm/kgLM) were confirmed using two-way analysis of variance (ANOVA). The ANOVA was supplemented with the Sidak post hoc test when the significance threshold was met. Differences in training characteristics between two age groups of active women and anthropometric characteristics between inactive and active groups were assessed using a Welch’s *t* test. The magnitude of the effect size was determined using the following criteria: d < 0.2 was considered to have no effect, 0.2 ≤ d < 0.5 was considered a small effect size, 0.5 ≤ d < 0.8 was considered a medium effect size, 0.8 ≤ d < 1.3 was considered a large effect size, and d ≥ 1.3 was considered a significant effect size [22]. The level of significance was set at 0.05. All statistical analyses were performed using SPSS version 26.0 (SPSS, Inc., Chicago, IL, USA).

## 3. Results

### 3.1. Characteristics of Participants

The active women in the present study engaged in running and also strength training. There was no significant difference in endurance and strength training volume between younger and older active women (Table 1). Age was similar between active and inactive groups, but the total body mass was higher in the inactive than in the active group (Table 2).

### 3.2. Muscular Strength Outcomes

The two-way ANOVA revealed that there was an age group effect (<50 and ≥50 yrs old; F(2146) = 40.5; *p* < 0.001) on peak torque (PT) for knee extensor muscles (Nm). However, the effect of the physical activity group (active or inactive) was not significant (Table 3). Conversely, the two-way ANOVA revealed that there was an age group effect (F(2146) = 44.1; *p* < 0.001) and physical activity group effect (F(2146) = 113.0; *p* < 0.001) on total body mass-adjusted PT values for knee extensor muscles (Nm/kg; Table 3). In the same direction, there was an age group effect (F(2146) = 36.9; *p* < 0.001) and a physical activity group effect (F(2146) = 6.1; *p* = 0.014) on lean mass-adjusted PT values for knee extensor muscles (Nm/kgLM; Table 3). The interaction effect for the three dependent variables was not significant.

Similar findings may be observed in the knee flexor muscles. The two-way ANOVA revealed that age group (<50 and ≥50 yrs old; F(2146) = 13.9; *p* < 0.001) and physical activity (F(2146) = 11.5; *p* < 0.001) had an impact on PT for knee flexor muscles (Nm; Table 4). In the same way, there was an age group effect (F(2146) = 16.3; *p* < 0.001) and physical activity group effect (F(2146) = 122.1; *p* < 0.001) on total body mass-adjusted PT values for knee flexor muscles (Nm/kg; Table 4). Finally, there was an age group effect (F(2146) = 9.1; *p* = 0.003) and physical activity group effect (F(2146) = 22.7; *p* < 0.001) on lean mass-adjusted PT values for knee flexor muscles (Nm/kgLM; Table 4). The interaction effect for the three dependent variables was not statistically significant.

### 3.3. Body Composition Outcomes

In terms of body composition, the two-way ANOVA revealed an age group effect (<50 and ≥50 yrs old; F(2146) = 5.8; *p* = 0.017) and physical activity effect (F(2146) = 225.1; *p* < 0.001) on fat mass (%) (Table 5). There was also an age group effect (<50 and ≥50 yrs old; F(2146) = 7.1; *p* = 0.009) on lean mass (kg), but there was no effect of the physical activity group on total lean mass. The interaction effect on fat mass and lean mass was not statistically significant.

## 4. Discussion

The loss of strength and muscle quality that occurs with the aging process, known as sarcopenia, is associated with loss of functional ability, higher risk of falls, and increased mortality risk. This is quite concerning, particularly for women, who have a higher incidence of sarcopenia and a longer life expectancy. Therefore, women may live longer with the functional disability caused by sarcopenia. Physical activity is one of the primary interventions recommended to prevent sarcopenia. In this context, the present study aimed to compare the muscular strength and lean mass of active and inactive women under or over 50 yrs old.

The main results of the present study were as follows: (a) absolute strength values for knee extensor muscles were significantly higher for <50 yrs than for ≥50 yrs but were similar between active and inactive groups; (b) absolute strength values for knee flexor muscles were significantly higher for <50 yrs than for ≥50 yrs and for the active group than for the inactive group; (c) total body mass-adjusted PT (Nm/kg) for knee extensor and flexor muscles were significantly higher for <50 yrs than for ≥50 yrs and for the active group than for the inactive group; (d) lower-limb lean mass-adjusted PT (Nm/kg) for knee extensor and flexor muscles were significantly higher for <50 yrs than for ≥50 yrs but similar between active and inactive groups; (e) the magnitude of the difference in strength levels (absolute or relative) between the <50 and ≥50 groups yrs was not different between the active and inactive groups; (f) lean mass was significantly higher for <50 yrs than for ≥50 yrs but was similar between active and inactive groups; and (g) fat mass percentage was significantly lower for <50 yrs than for ≥50 yrs and for active than for inactive group.

We initially hypothesized that active women would be stronger than inactive women in both age groups. Although this was confirmed for muscular strength values relative to body mass, it was not proven for knee extensor muscle absolute strength. We will discuss possible explanations for this result below. We also hypothesized that muscle quality would be better among active women, which was confirmed in the study. Finally, the study confirmed the initial hypothesis that the decline in strength with aging would be similar in active and inactive adults.

The first interesting result of the present study was that there was no significant difference between active and inactive groups in terms of absolute strength values for knee extensor muscles and lean mass. This lack of significance occurred despite the active group’s involvement in strengthening exercises for more than 150 min per week. This result appears surprising because trained women are expected to have more strength and lean mass. However, the inactive group had a higher total body mass and BMI than the active group, which is associated with increased muscle mass and absolute strength [23]. Furthermore, the knee extensor muscle is an antigravity muscle that is particularly active in daily activities, such as climbing stairs and getting up from a chair [24,25,26]. Therefore, knee extensor muscles in overweight women are overloaded and strengthened during these activities. Considering that individuals with higher BMI would be expected to have more strength [27,28], the physical activity habits of the active women (and those with lower BMI) attenuated the predicted differences in strength.

Despite having a lower BMI, the active group had considerably higher absolute values for knee flexor muscles than the inactive group. This finding indicates that the higher load transferred by the inactive group while doing daily living activities is inadequate to induce knee flexor muscle strengthening, most likely because knee flexor muscles, unlike knee extensor muscles, are not antigravity [29]. Therefore, unlike the knee extensor muscles, it appears that the knee flexor muscles require particular strengthening exercises to be strengthened.

In terms of muscle strength relative to total body mass, the active group had significantly greater values than the inactive group for both muscular groups evaluated (knee flexors and extensors). Because muscular strength related to total body mass is a better predictor of physical function than absolute muscle strength [30], these findings are more practical than absolute strength data. These findings were consistent with previously published data comparing the strengths of women with varying BMIs [31].

An interesting result can be observed when the aging effect on strength levels is considered. The <50-year-old group had higher strength levels than the ≥50-year-old group for active and inactive women, indicating that although strengthening exercises improve strength, the adverse effects of aging overlap, even in the absence of chronic disease. The most interesting result was that the magnitude of the difference in strength across age groups was the same for inactive and active women (no interaction effects), indicating that the result of aging is the same regardless of physical activity level. The volume of strength and aerobic training was not different between the two age groups; therefore, maintaining the training volume, strength loss occurs only as a result of the aging process [32].

Different results are typically observed for maximum oxygen uptake ($\dot{V}$O_2_max), another measure of functional ability and mortality risk. Hawkins et al. [33] revealed that $\dot{V}$O_2_max declines at a rate similar to or more than expected in inactive adults. Burtscher et al. [34] later demonstrated that the apparent more accelerated $\dot{V}$O_2_max drop among athletes as they age results from less training and the gradual aging-related $\dot{V}$O_2_max decline. In the same vein, the present study’s findings suggest that when training volume is maintained throughout time, the loss in muscular strength is comparable across active and inactive women, with the advantage that active women have higher strength relative to body mass at any age.

Finally, muscular strength has been represented in total lean mass, indicating strength per unit muscle mass [13], which measures muscular quality. The results revealed that the active group has higher muscular quality in the knee flexor and extensor muscles. The inactive group had higher fat mass than the active group, and it has previously been demonstrated that fat infiltration of muscle is associated with total body fat [35]; therefore, it is reasonable to assume that the inactive women in the present study have more fat infiltration in the muscle mass, which contributes to poor muscle quality. However, it is important to note that fat infiltration in muscle mass is not limited to sedentary or obese women. Adipose inflammation causes fatty infiltrations in skeletal muscles throughout aging [36], which can be observed in the present study by worse muscular quality in the ≥50-year-old group compared with the <50-year-old group for both groups (inactive and active). Interestingly, although active women had better muscle quality than inactive women in both age groups studied, the habit of performing physical activity did not mitigate the magnitude of the worsening, indicating that loss of muscle quality is one of the aging characteristics.

## 5. Limitations

This study has several limitations. This is a cross-sectional study. Therefore, it compares groups of varying ages and physical activity levels; however, to explore the effect of aging, the authors suggest that longitudinal studies be developed. Furthermore, the data on the participants’ strength and resistance training routine were subjective data, since they answered a questionnaire about it, and the answers may have been influenced by the participants’ memory and sincerity. Furthermore, the levels of physical activity of the participants who composed the active group were very different. The level of physical activity was classified according to the IPAQ questionnaire, and, among those who were classified as active, there were some who performed at the lower limit to be classified as active, and there were other women who performed much more weekly physical activity (2 or 3 times more). This fact can be considered a bias because both the amount and intensity of physical activity show a dose–response relationship with lower-limb strength [37].

## 6. Conclusions

In conclusion, both younger and older active women who engage in physical activities (aerobic and strength exercises) have higher thigh muscle strength and muscle quality than inactive women. Furthermore, the difference in muscle strength, mass, and quality between younger and older women is similar for active or inactive women. Considering that skeletal muscle mass and strength decline with age and muscular weakness is associated with frailty and a higher mortality risk, preventive strategies are necessary for health and well-being. The results of the present study suggest that continuing an aerobic and strengthening training routine is a viable choice for improving muscular strength and quality in both young and old women.

## Figures and Tables

**Table 1:** Characteristics of training habits from an active group.

	Active Women (<50 yrs)	Active Women (≥50 yrs)	*p* Value	Effect Size	Power
Endurance training (min/week)	273.4 ± 130.1	345.6 ± 207.1	0.080	0.41	0.59
Strength training (min/week)	161.5 ± 67.3	185.8 ± 120.7	0.326	0.24	0.30

Data are presented as mean ± standard deviation.

**Table 2:** Characteristics of the participants.

Variables	Age Group	Active Group (n = 85)	Inactive Group (n = 62)	ANOVA	F	*p* Value	Effect Size	Power
Age (yrs)	<50 yrs	35.8 ± 7.0	41.1 ± 6.1	Age group	365.7	<0.001	0.719	1.0
	≥50 yrs	60.6 ± 7.2	57.6 ± 4.2	Physical activity group	1.1	0.292	0.008	0.2
				Interaction	14.8	<0.001	0.094	0.9
Total body mass (kg)	<50 yrs	60.3 ± 6.5	81.5 ± 13.4	Age group	0.0	0.988	0.000	0.1
	≥50 yrs	59.1 ± 9.7	82.8 ± 9.9	Physical activity group	183.4	<0.001	0.562	1.0
				Interaction	0.6	0.454	0.004	0.6
Height (m)	<50 yrs	1.63 ± 0.05	1.60 ± 0.06	Age group	13.4	<0.001	0.086	0.9
	≥50 yrs	1.57 ± 0.06	1.59 ± 0.04	Physical activity group	0.1	0.713	0.001	0.1
				Interaction	6.0	0.015	0.041	0.7

Note. Data are presented as mean ± standard deviation.

**Table 3:** Mean and standard deviation values for knee extensor muscle peak torque (PT) (Nm), PT adjusted by total body mass (Nm/kg), and PT adjusted by lower-limb lean mass (Nm/kgLM) for each age group and physical activity group.

Variables	Age Group	Active Group (n = 85)	Inactive Group (n = 62)	ANOVA	F	*p* Value	Effect Size	Power
PT (Nm)	<50 yrs	128.4 ± 24.0	123.4 ± 28.5	Age group	40.5	<0.001	0.221	1.000
	≥50 yrs	102.0 ± 23.8	97.7 ± 19.3	Physical activity group	1.3	0.255	0.009	0.206
				Interaction	0.01	0.927	0.000	0.051
PT (Nm/kg)	<50 yrs	2.1 ± 0.4	1.5 ± 0.3	Age group	44.1	<0.001	0.230	1.000
	≥50 yrs	1.7 ± 0.3	1.2 ± 0.2	Physical activity group	113.0	<0.001	0.440	1.000
				Interaction	0.4	0.523	0.003	0.097
PT (Nm/kgLM)	<50 yrs	3.2 ± 0.5	3.0 ± 0.5	Age group	36.9	<0.001	0.205	1.000
	≥50 yrs	2.7 ± 0.5	2.5 ± 0.4	Physical activity group	6.1	0.014	0.041	0.694
				Interaction	0.1	0.014	0.000	0.051

Note. Effect size, η^2^p; power, 1 − β; PT, peak torque. Data are presented as mean ± standard deviation.

**Table 4:** Mean and standard deviation values for knee flexor muscle peak torque (PT) (Nm), PT adjusted by total body mass (Nm/kg), and PT adjusted by lower-limb lean mass (Nm/kgLM) presented by age group and physical activity group.

Variables	Age Group	Active Group (n = 85)	Inactive Group (n = 62)	ANOVA	F	*p* Value	Effect Size	Power
PT (Nm)	<50 yrs	69.0 ± 14.7	58.0 ± 15.3	Age group	13.9	<0.001	0.890	0.969
	≥50 yrs	57.1 ± 16.3	50.6 ± 14.9	Physical activity group	11.5	<0.001	0.750	0.921
				Interaction	0.8	0.384	0.005	0.140
PT (Nm/kg)	<50 yrs	1.15 ± 0.25	0.71 ± 0.16	Age group	16.3	<0.001	0.103	0.980
	≥50 yrs	0.97 ± 0.23	0.61 ± 0.17	Physical activity group	122.1	<0.001	0.461	1.000
				Interaction	1.2	0.263	0.009	0.201
PT (Nm/kgLM)	<50 yrs	1.7 ± 0.3	1.4 ± 0.3	Age group	9.1	0.003	0.060	0.851
	≥50 yrs	1.5 ± 0.4	1.3 ± 0.3	Physical activity group	22.7	<0.001	0.137	0.997
				Interaction	0.8	0.385	0.005	0.139

Note. Effect size, η^2^p; power, 1 − β; PT, peak torque. Data are presented as mean ± standard deviation.

**Table 5:** Mean and standard deviation values for body composition presented by age group and physical activity group.

Variables	Age Group	Active Group (n = 85)	Inactive Group (n = 62)	ANOVA	F	*p* Value	Effect Size	Power
Fat mass (%)	<50 yrs	29.4 ± 7.1	46.5 ± 5.8	Age group	5.8	0.017	0.039	0.667
	≥50 yrs	31.4 ± 9.6	50.2 ± 4.4	Physical activity group	225.1	<0.001	0.612	1.000
				Interaction	0.5	0.457	0.004	0.115
Total lean mass (kg)	<50 yrs	39.6 ± 3.6	40.8 ± 6.1	Age group	7.1	0.009	0.047	0.755
	≥50 yrs	37.5 ± 3.9	38.9 ± 4.3	Physical activity group	3.3	0.074	0.022	0.432
				Interaction	0.1	0.842	<0.001	0.055

Note. Effect size, η^2^p; power, 1 − β; PT, peak torque. Data are presented as mean ± standard deviation.

## Data Availability

We will make available the data upon request.
